# Correlation between progression of left ventricular mass and atherosclerosis of carotid and coronary arteries in patients with type 2 diabetes mellitus under optimal medical treatment: a long-term CMR study

**DOI:** 10.1186/1532-429X-15-S1-E37

**Published:** 2013-01-30

**Authors:** Tingting Xiong, David Jean Winkel, Eike Nagel, Nikolaus Tiling, Ashraf Hamdan, Rolf Gebker, Eckart Fleck, Ursula Plöckinger, Sebastian Kelle

**Affiliations:** 1 Internal Medicine/Cardiology, German Heart Institute Berlin, Berlin, Germany; 2 Interdisziplinäres Stoffwechsel-Centrum, Charité-Universitätsmedizin Berlin, Campus Virchow-Klinikum, Berlin, Germany; 3 Division of Imaging Sciences and Medical Engineering, King's College London, London, UK; 4 Chaim Sheba Medical Center, Tel Hashomer, Sackler Faculty of Medicine, Tel-Aviv University, Tel-Aviv, Israel

## Background

Increased left ventricular (LV) mass is a strong predictor of cardiovascular events and mortality and is common among patients with type 2 diabetes. Carotid intima-media thickness (CIMT) is commonly used to monitor progression of coexisting atherosclerosis. However, the relationship between the progression of LV mass and vascular changes in the carotid and coronary arteries remains poorly understood. The purpose of this study was to investigate that relationship in patients with type 2 diabetes.

## Methods

Cardiovascular magnetic resonance (CMR) imaging was performed at baseline and at 2-year follow-up in 95 patients with type 2 diabetes (57 men, mean age 61±9 years), of whom 86 had adequate image quality for common carotid artery and 37 for coronary artery vessel wall measurements. LV-septal and lateral wall thickness was assessed and LV mass index (LVMI) was determined by indexing LV mass to body surface area. For both vascular territories lumen area and total vessel area were assessed and vessel wall area, wall thickness and vessel wall ratio (vessel wall area/body surface area) were calculated. CIMT was measured by ultrasound.

## Results

All patients were under optimal medical treatment. At 2-year follow-up HbA1c significantly improved (HbA1c at baseline 7.88±1.76% vs. HbA1c at follow up 6.86±0.83% (p<0.001)) Blood pressure was normotensive at baseline (mean 128±13/73±10 mmHg); pulse pressure had not changed after 2 years of follow-up (p=0.292). LVMI and septal thickness were significantly higher at the 2-year follow-up (61.4±14.8 vs. 66.1±17.1 g/m^2^ and 10.7±2.0 vs. 11.5±1.9 mm, both p<0.001). Vessel wall area, wall thickness and vessel wall ratio of carotid and coronary artery remained unchanged. CIMT remained unchanged. Carotid vessel wall thickness at baseline was correlated to baseline LVMI (r=0.344, p=0.001) and septal thickness (r=0.406, p<0.001), but not to increment of LVMI (p = ns).

## Conclusions

LVMI and septal wall thickness showed a significant increment during a 2 year period, while vessel wall characteristics of carotid and coronary artery assessed by CMR remained unchanged. Interestingly, CIMT also remained unchanged over 2 years. These findings suggest that the increased risk of cardiovascular events in type 2 diabetes is possibly to be more attributed to increment of LV mass than to atherosclerotic vascular changes.

## Funding

none

